# Situs inversus: an interesting case of spontaneous splenic rupture

**DOI:** 10.1093/jscr/rjac033

**Published:** 2022-02-19

**Authors:** Darius Dastouri, William McSweeney, Suntharalingam Sivananthan

**Affiliations:** Department of General Surgery, Hervey Bay Hospital, Queensland Health, Hervey Bay, Queensland 4655, Australia

## Abstract

Situs inversus is described as exact mirroring of the normal anatomical arrangement of the major visceral organs. Polysplenia is a congenital anomaly associated with situs inversus and causes various splenic abnormalities. This case discusses a 62-year-old female who presented to the emergency department with hypotension and abdominal pain. Commuted tomography reveals situs inversus and a lobulated mass in the right upper quadrant consistent with a splenic rupture intraoperatively. This is the first reported case of a spontaneous splenic rupture in a patient with situs inversus. This case highlights the rarity of splenic injuries in situs inversus and the unique anatomical challenges that surgeons are faced with intraoperatively in a high-pressure environment.

## INTRODUCTION

Normal anatomical orientation of the major viscera is termed situs solitus [1]. Situs inversus (SI) is a rare congenital anomaly where the major abdominal viscera are mirrored from their normal anatomical arrangement [1, 2]. The prevalence of SI ranges between 1:6000 and 1:10 000 with approximately one-third being associated with the recessive genetic condition, Primary Ciliary Dyskinesia [1, 2]. SI can be partial or total, and the term situs ambiguous refers to the spectrum seen in the anatomical variation between the cardiac atria and major viscera [1, 3]. SI is commonly discovered in the first few years of life as 5–10% of cases are associated with congenital heart defects or other gastrointestinal anomalies [3–5].

Heterotaxy describes the variety of syndromes associated with situs ambiguous and is often used in reference with polysplenia syndrome [1, 4, 6]. Polysplenia is one of the congenital anomalies associated with approximately 20% of SI cases resulting in the development of 2 to 16 spleens [4]. Three types of splenic anomalies are described: asplenia, multiple splenunculi of varying sizes and a normal sized spleen in the right upper abdomen [1]. This case describes the first reported case of a spontaneous splenic rupture in a patient with SI and polysplenia.

## CASE REPORT

A 62-year-old female presented to the emergency department with a 1-day history of sudden onset generalized abdominal pain, anorexia and vomiting. She reported passing wind but not opening her bowels during this period. She denied any infective symptoms or thoracoabdominal trauma leading up to her presentation. Her medical history is relevant for SI totalis, mechanical mitral valve replacement for which she takes regular coumadin and a previous laparoscopic tubal ligation. Her vital signs on arrival showed a blood pressure of 96/67 mmHg, heart rate of 82, respiratory rate of 18 and was afebrile. On examination she had generalized abdominal tenderness and right upper quadrant peritonism, with scant bowel sounds. Her heart sounds were dual with clear lung fields on auscultation. Initial laboratory investigations revealed an international normalized ratio (INR) of 3.4, haemoglobin of 119, haematcrit of 0.35, platelet count of 120, white blood cell count of 9900, creatinine 78, lipase 19. She had no significant acid–base disturbances.

**Figure 1:**
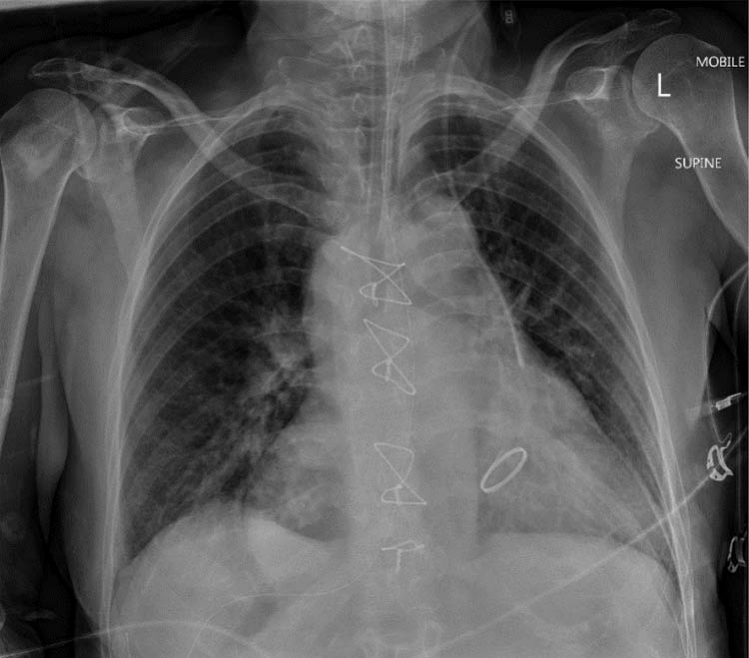
Mobile anteroposterior chest film. Demonstrates the nasogastric tube right of midline in stomach and the left internal jugular central line left of midline in the superior vena cava.

**Figure 2:**
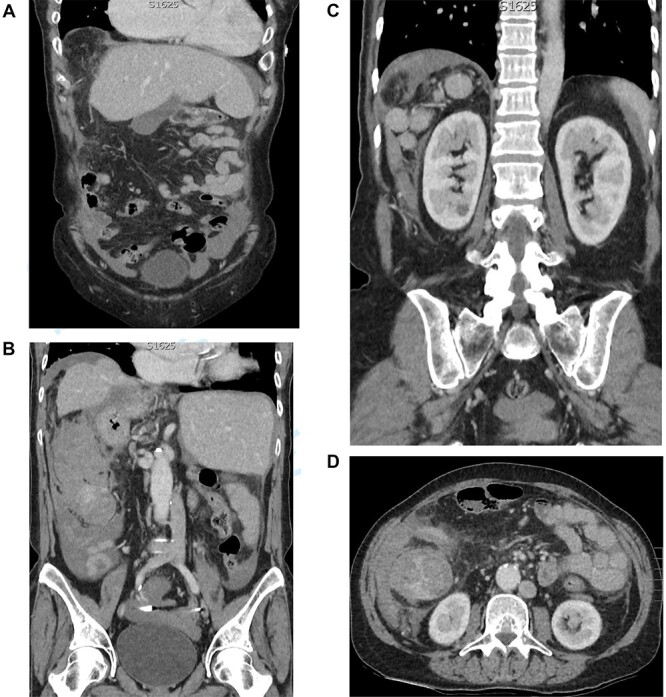
(**A**) CT coronal cross section demonstrating a left-sided liver and gallbladder. (**B**) CT coronal cross section demonstrating a right-sided stomach and abdominal aorta, left-sided inferior vena cava and the heterogenous lobulated mass with free fluid. (**C**) CT coronal cross section demonstrating the five splenunculi. (**D**) CT axial cross section demonstrating the 130 mm heterogenous lobulated mass in the right abdomen

Figure 1 shows the plain film chest X-ray. Commuted tomography (CT) with intravenous contrast of the abdomen and pelvis confirmed SI totalis (Fig. 2). Polysplenia with five splenunculi, a lobulated 130 mm mass with heterogenous contrast enhancement and free fluid were noted in the right upper quadrant on CT (Fig. 2). Upon return from CT the patient became tachycardiac and hypotensive to 62/44 mmHg for which she was fluid responsive to 2 L IV crystalloid. Repeat haemoglobin was now 109 with a lactate of 0.9 and no significant acid–base disturbance. Group and Save, IV 10 mg vitamin K and 2300 IU of prothrombinex-VF were ordered, and the general surgical team was consulted urgently.

**Figure 3:**
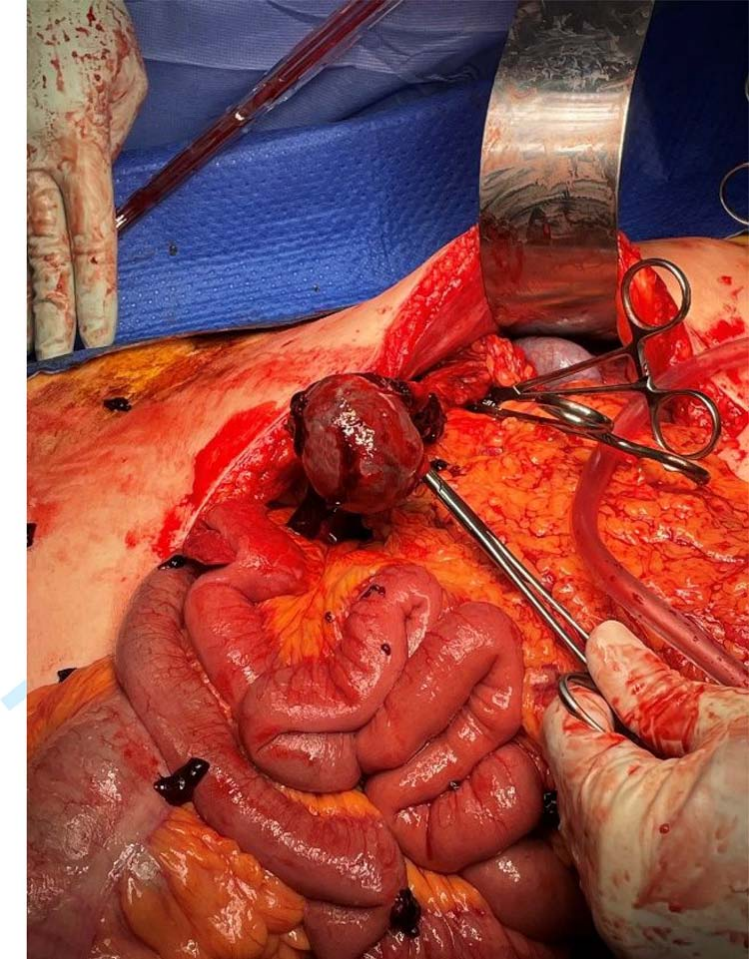
Intra-operative laparotomy showing accessory spleen before resection. Omentum has been dissected away and feeding vessels controlled.

**Figure 4:**
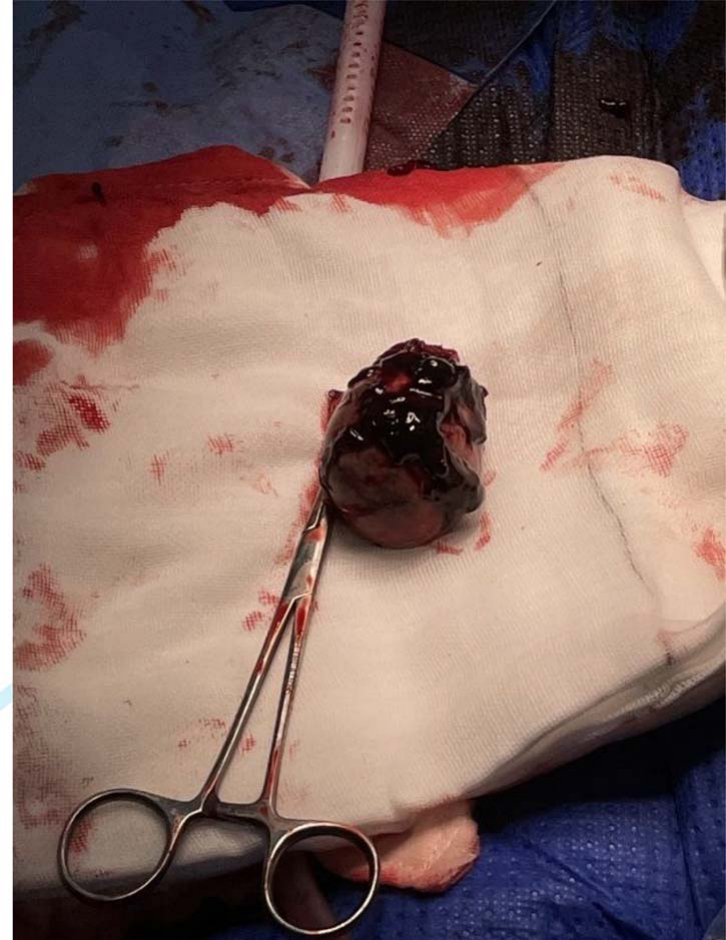
Accessory spleen post resection showing closely adhered clot.

The patient proceeded to a diagnostic laparoscopy with a blood pressure of 109/72 mmHg and a heart rate of 105. She had received one unit of PRBC and her repeat INR was 1.4. Diagnostic laparoscopy showed four quadrant haemoperitoneum and conversion to laparotomy was performed. Approximately 850 ml of blood was suctioned from the abdomen and the omentum was noted to be wrapped around a clotted splenunculi in the right abdomen (Fig. 3). Remaining splenunculi and intra-abdominal organs were unremarkable. Partial splenectomy and omentectomy of the affected splenuculus and omentum were performed with a thunderbeat surgical energy device. Haemostasis was further achieved with 0 vicryl ties and surgical absorbable haemostat. Prophylactic appendicectomy was performed. Histology confirmed a 45×40 mm accessory spleen with a 15×13 mm tear (Fig. 4).

Postoperatively the patient was monitored in the intensive care unit and had a haemoglobin of 98. Coumadin was withheld and the patient was commenced on an IV heparin infusion and remained within therapeutic range. On postoperative Day 4, the patient had fresh blood in the drain and normal haemodynamics. Biochemistry showed a haemoglobin drop from 84 to 61 (haematocrit 0.26–0.19), INR of 1.2 and a supratherapeutic APTT of 120. Exploratory laparotomy revealed four quadrant haemoperitoneum and clot in the right abdomen and pelvis from generalized oozing from the right paracolic gutter. Haemostasis was achieved with abdominal packs and pressure with no further active bleeding. The patient was again returned to the intensive care for observation and the IV heparin infusion was recommenced the following morning.

The patient suffered no further bleeding and coumadin was recommenced on postoperative Day 4. The patient was discharged home on Day 15 with a haemoglobin of 106 and therapeutic INR of 2.6. She has since been followed up in the surgical outpatient’s department and is doing well.

## DISCUSSION

SI is a rare congenital abnormality resulting in the transposition and mirroring of major organs [1]. Most diagnoses are made in neonates and infants for work up of symptomatic congenital heart disease or gastrointestinal symptoms but are usually incidentally found on imaging in adults [1–3]. Polysplenia is seen in 20% of cases of SI and belongs to its own group of heterotaxic syndromes [4].

To the best of the authors’ knowledge, this is the first documented case of a spontaneous splenic rupture in a patient with SI and polysplenia. It is likely that the patient’s prior anticoagulation may have exacerbated microvascular trauma in the spleen resulting in an insidious injury causing spontaneous rupture and haemorrhage. This case presented an anatomical and postoperative challenge given the patients anticoagulation for a mitral valve replacement. Laparoscopy in patients with SI poses an unusual anatomical challenge due to mirroring of the organs and major vessels requiring consideration for port placement. In this case, the team quickly converted to laparotomy to increase exposure to aid our anatomical landmarks to rapidly control haemorrhage. The presence of multiple splenunculi did not pose any surgical challenge.

In the scenario of intra-abdominal haemorrhage and SI, the authors recommends early conversion to laparotomy and the attendance of a senior surgeon to help navigate anatomical challenges in a high-pressure environment.

## CONFLICT OF INTEREST STATEMENT

None declared.

## INFORMED CONSENT

Informed consent was obtained preoperatively.
